# Effect of the maternal sleep disturbances and obstructive sleep apnea on feto‐placental Doppler: A systematic review

**DOI:** 10.1111/jsr.14460

**Published:** 2025-01-15

**Authors:** Marco La Verde, Maria Maddalena Marrapodi, Marica Palma, Davide Pisani, Diana Russo, Vincenzo Ronsivalle, Marco Cicciù, Giuseppe Minervini

**Affiliations:** ^1^ Department of Woman, Child and General and Specialized Surgery, Obstetrics and Gynecology Unit University of Campania “Luigi Vanvitelli” Naples Italy; ^2^ Department of Woman, Child and General and Specialist Surgery University of Campania “Luigi Vanvitelli” Naples Italy; ^3^ Multidisciplinary Department of Medical‐Surgical and Dental Specialties, Oral Surgery Unit University of Campania “Luigi Vanvitelli” Naples Italy; ^4^ Department of Biomedical and Surgical and Biomedical Sciences Catania University Catania Italy; ^5^ Saveetha Dental College and Hospitals, Saveetha Institute of Medical and Technical Sciences (SIMATS) Saveetha University Chennai India; ^6^ Multidisciplinary Department of Medical‐Surgical and Dental Specialties University of Campania “Luigi Vanvitelli” Naples Italy

**Keywords:** cerebroplacental ratio, feto‐placental Doppler, obstructive sleep apnea, sleep‐disordered breathing, umbilical artery, uterine artery Doppler

## Abstract

Literature evidenced an association of maternal sleep disturbances and maternal obstructive sleep apnea with significant obstetric complications. Moreover, the maternal sleep disturbances effect on feto‐placental circulation had not been extensively examined. Our objective is to explore the possible maternal sleep disturbances impact on the feto‐placental indices evaluated through the Doppler study. A systematic review of the following databases was performed: PubMed, EMBASE, Cochrane Library and Google Scholar from the beginning to June 2024. Only studies that enrolled pregnant women with signs and symptoms of maternal sleep disturbances or obstructive sleep apnea diagnosis, which analysed the feto‐placental Doppler parameters, were considered eligible (PROSPERO ID: CRD42024553926). We included a total of four studies with 1715 cases of pregnant women. Various instrumental and non‐instrumental diagnostic methods were adopted for detection of maternal sleep disturbances. The ultrasound exam was performed mainly in the third trimester of pregnancies, and all the studies explored the uterine Doppler parameters. Only two studies explore the foetal Doppler parameters. Only one study disclosed that maternal sleep disturbances are related to altered uterine Doppler indices with probable placental dysfunction. This review did not evidence a significant influence of maternal sleep disturbances and obstructive sleep apnea on foetal Doppler indices. Moreover, one large prospective study showed a possible impact of maternal sleep disturbances on uterine Doppler with a potential impairment of the placentation function. Additional studies with detailed data and larger samples are needed to throw light on this relationship and its impact on the foetal outcomes.

## Graphical abstract

Literature evidenced an association of maternal obstructive sleep apnea (OSA) with significant obstetric complications. Moreover, the OSA and its effect on feto‐placental circulation had not been extensively examined. Our objective is to explore the possible impact of OSA on the feto‐placental indices evaluated through the Doppler study. A systematic review of the following databases was performed: PubMed, EMBASE, Cochrane Library and Google Scholar from the beginning to June 2024. Only studies that enrolled pregnant women with signs and symptoms of OSA, which analysed the feto‐placental Doppler parameters, were considered eligible (PROSPERO ID: CRD42024553926). We included a total of four studies with 1715 cases of pregnant women. Various instrumental and non‐instrumental diagnostic methods were adopted for OSA detection. The ultrasound exam was performed mainly in the third trimester of pregnancies, and all the studies explored the uterine Doppler parameters. Only two studies explore the foetal Doppler parameters. Non‐statistical difference was found in the foetal Doppler indices in the pregnant with OSA. Only one study disclosed that OSA is related to altered uterine Doppler indices with probable placental dysfunction. This review did not evidence a significant influence of maternal OSA on foetal Doppler indices. Moreover, one large prospective study showed a possible OSA impact on uterine Doppler with a potential impairment of the placentation function. Additional studies with detailed data and larger samples are needed to throw light on this relationship and its impact on the foetal outcomes.

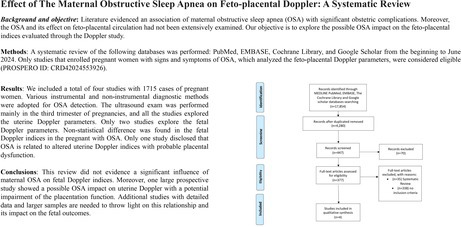

## 1 INTRODUCTION

Maternal sleep disturbance is a common occurrence in pregnancy, with significant implications for maternal and foetal health. The common sleep disturbances reported from different studies include poor sleep quality, insomnia, and diagnosed conditions like obstructive sleep apnea (OSA) (Lu et al., 2021). Evidence shows maternal sleep disturbances are related to gestational diabetes mellitus (GDM), hypertension, and pre‐eclampsia or foetal growth (Sanapo et al., 2024). Consequently, early diagnosis and management are necessary (Ding et al., 2014). These findings highlighted the importance of addressing maternal sleep health in prenatal care. OSA is one of the most frequent types of sleep‐disordered breathing, and it is featured in several hypoxia events due to upper airway obstruction during sleep (Sahin et al., 2008). OSA is common in pregnancy, especially during the third trimester (Silvestri & Aricò, 2019). Snoring during pregnancy was estimated at about 25% of the pregnant women (Facco et al., 2017). The real prevalence of OSA in pregnant women is not well known because a great number of studies are based on clinical symptoms or on the compilation of questionnaires rather than the diagnostic gold‐standard, polysomnography (PSG; Johns et al., 2022). Although the questionnaires do not require a specific clinical setting for their compilation and are easier to administer than PSG, they do not adequately diagnose OSA in pregnancy (Chen et al., 2012). Pregnant weight increase, the oedema of the upper airways and hormonal influence may cause OSA onset in pregnant women (Uzunçıbuk et al., 2023; Pien et al., 2005; Tantrakul et al., 2017). Clinical manifestations of this chronic pathology are snoring, gasping, witnessed apnea, daytime sleepiness and fatigue, and any categories are more susceptible of the OSA consequence (Bourjeily et al., 2012; Almeida et al., 2023). In patients with OSA, conservative management is implemented when the temporomandibular joint disorder is present (Minervini, Marrapodi, & Almeida Cicciù, 2023; Solak et al., 2009). The treatment of this disorder focuses on reducing the symptoms and enhancing the functional capacity (LeResche et al., 2005; Langaliya et al.,  2023). Other researchers reported the negative impact of OSA on pregnancy, which included an increased risk of GDM, low birth weight, gestational hypertension and preeclampsia (Franklin et al., 2000; Louis et al., 2014). The mechanism by which OSA contributes to adverse pregnancy outcomes is not well known yet (Ravishankar et al., 2015; Robertson et al., 2019). It has been hypothesised that periods of maternal hypoxia during sleep cause a condition of placental hypoperfusion and hypoxygenation with an impairment of its function (Ravishankar et al., 2015; Robertson et al., 2019). In support of this hypothesis, an altered pattern of placental function markers has been shown in pregnant women with OSA (Bourjeily et al., 2015). A non‐invasive and reliable indicator of feto‐placental unit function is Doppler ultrasound (Ashwal et al., 2022; La Verde et al., 2023). Doppler study of the umbilical artery (UA), middle cerebral artery (MCA) and uterine arteries (UtA) provides information about the placental perfusion status and foetal well‐being, and ultrasound represents an essential tool to perform the follow‐up of foetal development and maternal–foetal health in pregnancy (Dunn et al., 2017; Khalil & Thilaganathan, 2017; La Verde, De Franciscis, et al., 2022). Pulsatility index is one of the most important parameters of the Doppler spectrum (Dunn et al., 2017; Khalil & Thilaganathan, 2017) – it indicates the impedance to blood flow of the arteries (Da Costa et al., 2005). There is an increase in blood flow resistances in the UtA and UA, and at the same time a reduction in vascular resistances in the MCA, in all situations of placental malfunction and foetal hypoxia to preserve the circulation of the noble foetal organs (brain, heart and adrenal gland; Flood et al., 2014). This mechanism of foetal autoregulation is called Brain spearing (Wladimiroff et al., 1986). A great deal of interest in OSA scientific research is due to the frequency and possible influence on pregnancy adverse events. Given the high prevalence of OSA, which may be contributing to adverse pregnancy outcomes, this becomes a focus of considerable research interest. Indeed, few studies have evaluated the association of maternal OSA with feto‐placental Doppler indices alterations despite the adverse impact of OSA on pregnancy outcomes reported in the existing literature. The present systematic review, therefore, has the primary purpose of critically assessing the available literature data on the effect of maternal OSA on feto‐placental Doppler indices.

## 2 MATERIALS AND METHODS

### 2.1 Eligibility criteria

Only studies that studied ultrasound Doppler indices in single‐pregnant women with signs and symptoms of OSA in pregnancy were considered eligible for this systematic review. We included patients with an OSA diagnosis or with a high risk of OSA evaluated with the Epworth Sleepiness Scale (ESS), Berlin Questionnaire (BQ) or Pittsburgh Sleep Quality Index (PSQI). Any type of study on OSA in pregnancy that did not focus on maternal–foetal Doppler indices was excluded from the review. No language or geographical limitations were placed.

### 2.2 Information sources

This systematic review was carried out according to the Preferred Reporting Items for Systematic Review and Meta‐analysis (PRISMA) guidelines (Liberati et al., 2009). Previously the protocol of our scientific papers was defined, and it was recorded on the International Prospective Register of Systematic Reviews (PROSPERO) database (Registration number CRD42024553926). A comprehensive, detailed search in the following databases was performed: PubMed, EMBASE, The Cochrane. Additional articles were identified by searching the reference lists from included studies and from Google scholars.

**FIGURE 1:**
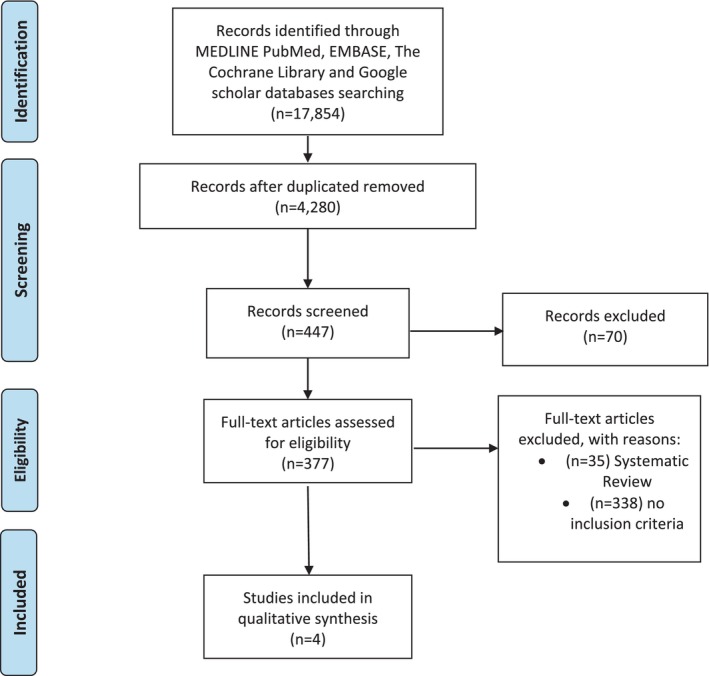
The PRISMA flowchart of the literature search.

### 2.3 Search strategy

A combination of MeSh and keywords, such as “maternal sleep disorders”, “sleep‐disordered breathing”, “foetal Doppler” and “Doppler ultrasound”, was used to search for publications aimed to our review. We selected every article published from the inception until 4 June 2024.

### 2.4 Study selection

Two authors (MP and DP) analysed the selected studies, excluding duplicates and those that did not consider the interaction between maternal sleep disorders and ultrasound Doppler parameters. Full‐text analysis was done for those articles found to fully meet the inclusion criteria. To be included, the studies reported: (1) single‐pregnancy; (2) the presence of signs and symptoms of maternal sleep disorders in pregnancy; (3) performance of the feto‐placental Doppler study in pregnant women in the second and third trimesters of gestation. The authors excluded all papers that did not meet these criteria. Whenever the two authors were uncertain about the validity of the study, they consulted a third author to clarify doubts (MLV).

### 2.5 Data extraction

Data extraction was performed by two authors (MP and DP) independently for the articles describing the type of study, first author, year of publication, country, diagnostic criteria for OSA, assessment of OSA, period's evaluation of feto‐maternal Doppler, age OSA/control, body mass index (BMI) OSA/control, number of OSA (based on method used)/control, total of subjects studied, number of subjects with OSA‐altered Doppler/control. One author (MLV) checked the consistency of data extraction after going through the entire data extraction process.

### 2.6 Risk of bias assessment

Two authors (MP and DP) have independently made an assessment of the risk of bias for the studies included in this systematic review, based on the Newcastle–Ottawa Scale modify form (Stang, 2010). In every study, several factors at the quality levels have been assessed under five independent domains: “study design and sample representativeness”, “sample technique”, “description of the OSA diagnostic technique”, “quality of the population description” and “incomplete outcome data” (Table S1). Any discrepancies between the reviewers were resolved by a third reviewer (MLV).

### 2.7 Outcome measures and data synthesis

The primary objective of this study was to evaluate the abnormal Doppler in pregnant women affected by maternal sleep disorders or OSA. We evaluated the number of subjects with maternal sleep disorders and the respective altered foetal Doppler. If reported, the foetal outcomes were evaluated. Data heterogeneity due to different settings, outcomes and OSA diagnostic tools made quantitative analysis impossible.

## 3 RESULTS

### 3.1 Study selection

Two authors screened the titles and abstracts of the 447 articles. Many papers were discarded because they did not focus on the relation between sleep breath disorders and maternal–foetal Doppler. To verify that the 377 eligible articles met the inclusion criteria established at the outset, they were examined in full text. Articles not meeting inclusion criteria were excluded. Where such doubts existed regarding its suitability to the study, the two authors resolved uncertainty by reaching a third author. At the end of the selection process, only four studies were considered eligible by all authors (Figure 1; Onslow et al., 2023; Robertson et al., 2020; Robertson et al., 2022; Tang et al., 2021).

### 3.2 Study characteristics and synthesis of the results

Table 1 summarises these studies regarding the diagnostic criteria, OSA assessment methods, period of feto‐maternal Doppler evaluation, and type of study design. Each article evaluated the symptoms and signs of OSA during pregnancy with different modalities. One case–control study (Onslow et al., 2023) and three prospective cohort studies were included in this review (Robertson et al., 2020; Robertson et al., 2022; Tang et al., 2021). All articles included have been published between 2010 and 2022. As Table 1 shows, only one study performed the OSA diagnosis with PSG (Onslow et al., 2023). The diagnostic criteria applied was an apnea–hypopnea index (AHI) ≥ 5 (Onslow et al., 2023). The other three studies investigated the OSA through non‐instrumental diagnostic methods as self‐administer questionnaires (Robertson et al., 2020; Robertson et al., 2022; Tang et al., 2021): BQ; ESS; and PSQI (Table 1). All pregnant women enrolled in the studies had performed an ultrasound test in the third trimester of pregnancy to study maternal–foetal Doppler according to the main ultrasound guidelines (Onslow et al., 2023; Robertson et al., 2020; Robertson et al., 2022; Tang et al., 2021); one study also included the second trimester of pregnancy (Robertson et al., 2022; Table 1). According to Table 2, the total number of subjects included in our revision was 1715. The subjects affected by OSA varied among the studies, ranging from 58 to 715 pregnant women. OSA diagnosis was based on various diagnostic criteria, for example, the ESS, BQ and AHI. The population analysed was heterogeneous in baseline characteristics (Table 2). The mean age in the studied population ranged between 27.42 ± 4.87 years and 31.6 + 3.7 years, as reported in Table 2. All pregnant women with OSA were overweight or obese, the lowest mean BMI of a patient was 24 kg m^−2^ (22.0–27.3 kg m^−2^), while the highest mean BMI was 42.33 ± 7.82 kg m^−2^. The study of Yafang Tang evidenced a strong correlation between OSA and the UtA Doppler modifications (Tang et al., 2021). They evidenced UtA Doppler alterations in 695 subjects affected by OSA (Tang et al., 2021). However, the other three studies did not show a relation between UtA Doppler alteration and OSA, reporting normal UtA Doppler values (Onslow et al., 2023;Robertson et al., 2020; Robertson et al., 2022). Only two studies included in the review explored the foetal Doppler and did not report foetal Doppler alterations (Robertson et al., 2020; Robertson et al., 2022).

**TABLE 1:** Characteristics of studies included in the systematic review.

First author	Year	Country	OSA diagnostic criteria	OSA assessment	Period's evaluation of feto‐maternal Doppler	Type of study
Tang	2010	Singapore	PSQI > 5	PSQI	III trimester	Prospective study
Robertson	2020	Australia	Severe if the woman answered “yes” to snoring ≥3 times per week, or answered “yes” to choking or gasping during sleep; and defined as mild if the woman answered “yes” to snoring but < 3 times per week	BQ	III trimester	Prospective observational cohort study
Robertson	2022	Australia	ESS > 11	ESS	II–III trimester	Prospective observational cohort study
Onslow	2022	USA	AHI ≥ 5	PSG	III trimester	Case–control study

AHI, apnea–hypopnea index; BQ, Berlin Questionnaire; ESS, Epworth Sleepiness Scale; OSA, obstructive sleep apnea; PSG, polysomnography; PSQI, Pittsburgh Sleep Quality Index.

**TABLE 2:** Characteristics of the studies on correlation between sleeping breathing disorders and feto‐maternal Doppler alterations.

First author	Age SBD/control	BMI SBD/control	Number of SBD (based on method used) /control	Total of subjects studied	SBD pregnant with foetal Doppler alteration	UtA‐PI
Tang	30.54 (5.0)	28.1 ± 4.8	715	916	NR	0.6 (0.6–0.8)^a^
Robertson	31.6 (3.7) 30.6 (3.9)	29.8 (27.2–34.4) 27.45 (24.6–30.5)	BQ > 5 *N* 163 BQ < 5 *N* 92	255	0	0.68 (0.64–0.72) 0.65 (0.59–0.70)^b^
Robertson	31.4 (4.4) 31.0 (4.0)	24.0 (22.0 27.3) 23.0 (20.7 27.2)	ESS > 11 *n* 58 ESS < 11,244	302	0	0.68 (0.65–0.71)^a^ 0.69 (0.65_0.74)^a^
Onslow	29.51 ± 5.58 27.42 ± 4.87	42.33 ± 7.82 38.42 ± 5.32	AHI > 5 *n* 89 AHI < 5 *n* 153	242	NR	1.15 ± 0.43 1.04 ± 0.32

AHI, apnea–hypopnea index; BMI, body mass index; BQ, Berlin Questionnaire; ESS, Epworth Sleepiness Scale; NR, not reported; SBD, sleeping breathing disorders; UtA‐PI, uterine artery‐pulsatility index.

^a^ III trimester values.

^b^ Mild symptoms group.

### 3.3 Risk of bias of included studies

Of the four included studies, three had a low risk of bias in three or more domains (Robertson et al., 2020; Robertson et al., 2022), and there was only one high risk of bias study (Dominguez et al., 2018). The risk of bias information for each domain in all studies is available in Table S2.

## 4 DISCUSSION

The main goal of this systematic review was to estimate the impact of maternal sleep disturbances on maternal–foetal Doppler indices. Our main findings did not show an association of maternal sleep disturbances with altered parameters of foetal blood flow (Robertson et al., 2020; Robertson et al., 2022). We only evidenced an increased resistance in UtA, as reported in one large prospective study (Tang et al., 2021). These changes may point to impairments in placental function and possible foetal hypoxia, thus contributing to adverse pregnancy outcomes in the OSA subgroup. Scientific literature lacks data about OSA pregnancies and placental negative impact. The observed modifications in UtA Doppler align with this hypothesis of impaired placental perfusion and foetal oxygenation in pregnant women affected by OSA (Tang et al., 2021). Robertson et al. (2020) evidenced no differences in results for feto‐placental Doppler indices, including UA pulsatility index and MCA pulsatility index, as well as UtA Doppler parameters between women with or without OSA symptoms (Robertson et al., 2020). They did not find evidence for the significant impact of OSA symptoms on feto‐placental circulation (Robertson et al., 2020). They supposed other mechanisms to explain negative outcomes in pregnancies complicated by OSA. Robertson et al. (2022) conducted another study where the effect of ESS‐assessed maternal sleep‐disordered breathing on parameters of feto‐placental Doppler in pregnancy was evaluated (Robertson et al., 2022). Also, this study did not find a correlation of ESS with feto‐placental Doppler indices, including MCA and UA (Robertson et al., 2022). Onslow et al. found no significant interaction of OSA with either UtA or angiogenic markers emerged, suggesting that OSA per se does not influence these specific vascular or placental factors among obese pregnant women (Onslow et al., 2023). They concluded that OSA did not impact on feto‐placental blood flow or angiogenic markers in obese pregnant women (Onslow et al., 2023). The literature is rich in significant associations linking sleep‐disordered breathing during pregnancy with hypertensive disorders developing in pregnancy (HDP) and with GDM (Sahin et al., 2008). Several studies prove a significant association between OSA, specifically obstructive sleep apnea, and these adverse pregnancy outcomes (Facco et al., 2017; Liu et al., 2019; Querejeta Roca et al., 2020). Facco et al. reported approximately double the adjusted odds ratios for both preeclampsia and HDP in women with OSA (Facco et al., 2017). Notably, this finding agrees with the review by Querejeta Roca et al. relative to OSA and HDP, particularly preeclampsia (Querejeta Roca et al., 2020). This review commented that the risk of HDP increases with increasing OSA severity, independent of association of severity symptoms (Querejeta Roca et al., 2020). According to a meta‐analysis by Liu, OSA is associated with HDP and GDM (Liu et al., 2019). OSA during pregnancy represents a critical risk factor for the development of HDP and GDM, but data about the exact mechanism are lacking. OSA treatment during pregnancy will improve the outcomes for both mother as well as the foetus (Abdullah et al., 2016; Alshadidi et al., 2023). Maternal oxygenation seems to be improved with continuous positive airway pressure (CPAP) therapy and parent education level (Dominguez et al., 2018). Several limitations are present in our review. First of all, only one study applied PSG as a diagnostic tool (Onslow et al., 2023). Three studies adopted non‐instrumental diagnostic methods, such as self‐administer questionnaires: BQ (Robertson et al., 2020); ESS (Robertson et al., 2022); and PSQI (Tang et al., 2021). Consequently, the majority of our cohort consisted of pregnant women with maternal sleep disturbances or identified as being at high risk for OSA based on clinical symptoms and screening tools. While this broader inclusion captures the spectrum of sleep‐disordered breathing in pregnancy, it inherently brings a variation into our findings in that results may not represent precisely the pathophysiological consequences of OSA. This is the limitation toward which important gaps in the available literature are highlighted, mainly in the few strong and targeted studies of OSA concerning foetal wellbeing via assessments through feto‐placental Doppler. Additionally, the population and the sample size differed among the studies included. The studies were also conducted in a broad spectrum of clinical setting so their findings would be relevant to many healthcare contexts. At least, considerable variables could influence pregnancy (Costa et al., 1998; Luciano et al., 2022; Roy‐Matton et al., 2011), and our review lacks homogeneous data collection across studies, including information on cardiotocography and neonatal outcomes (Bourjeily et al., 2020; La Verde, Riemma, et al., 2022), to enable more accurate comparisons and conclusions. Consequently, our findings are not generalisable. These differences could explain the different results of the UtA Doppler findings. At least the studies exploring the foetal Doppler are insufficient, and the total included population is inadequate to consider their findings conclusive (Robertson et al., 2020; Robertson et al., 2022). These limitations underline the urgent need for knowledge deficiencies in prospective studies with uniform diagnostic protocols in delineating the impact of OSA on feto‐placental circulation with modern imaging modalities, such as Doppler ultrasonography. Our review had different strengths, considering that it represents the first review to rigorously explore the literature about the impact of maternal OSA on foetal–maternal Doppler parameters. Our focus on these gaps in the literature and the call for further research with standardised diagnostic criteria will make the case for further investigation of this critical area, underlining the practical relevance of our findings on clinical practice.

## 5 CONCLUSIONS

In conclusion, this systematic review explores the effects of OSA and maternal sleep disturbances on Doppler parameters of the feto‐placental circulation for the first time. In our review, no significant relation between maternal sleep disturbances and changes in foetal blood flow was found. However, an altered UtA resistance has been observed in one study and indicates a possible impact on placental function. These possible links and their pathophysiological mechanisms need to be refined in future studies, with uniform protocols and larger samples aiming to explore OSA‐related placental dysfunction.

## INFORMED CONSENT STATEMENT

The patient signed an Informed Consent and agreed to the publication of anonymous data.

## AUTHOR CONTRIBUTIONS

**Marco La Verde:** Conceptualization; methodology; writing – original draft; investigation. **Maria Maddalena Marrapodi:** Data curation; software; investigation; writing – original draft. **Marica Palma:** Data curation; investigation; formal analysis. **Davide Pisani:** Conceptualization; investigation; validation; methodology. **Diana Russo:** Formal analysis; funding acquisition; visualization. **Vincenzo Ronsivalle:** Visualization; formal analysis. **Marco Cicciù:** Supervision; visualization; writing – review and editing. **Giuseppe Minervini:** Writing – review and editing; writing – original draft; conceptualization.

## FUNDING INFORMATION

This research received no external funding.

## CONFLICT OF INTEREST STATEMENT

The authors declare no conflicts of interest.

## Supporting information


**TABLE S1.** Modified Newcastle–Ottawa scale items.

**TABLE S2.** Risk of bias assessment.

## Data Availability

The data that support the findings of this study are available from the corresponding author upon reasonable request.
