# Supplementary material for: Effect of the maternal sleep disturbances and obstructive sleep apnea on feto‐placental Doppler: A systematic review
Source: J Sleep Res. 2025 Jan 15;34(4):e14460. doi: 10.1111/jsr.14460 (PMC12215232; doi:10.1111/jsr.14460)
Supplement: Supplementary file 1 — TABLE S1. Modified Newcastle–Ottawa scale items. [file JSR-34-e14460-s001.docx]

**(1) Study design and sample representativeness:**

1 point: Study design involved a control group, sample size was greater than or equal to 100.

0 points: Uncontrolled study, sample size less than 100 participants.

**(2) Sampling technique:**

1 point: Patients recruited consecutively or randomly (randomization criteria clarified).

0 points: Potential convenience sampling or unspecified sampling technique.

**(3) Description of the OSA diagnostic technique:**

1 point: The authors provided a comprehensive description of the polysomnography equipment, setting, and adopted technique.

0 points: The study did not report adequate information on the polysomnography evaluation or did not adopt polysomnography.

**(4) Quality of population description:**

1 point: The study reported a clear description of the population with proper measures of dispersion (e.g., mean, standard deviation).

0 points: The study did not report a clear description of the population, incompletely reported

descriptive statistics or did not report measures of dispersion**.**

**(5) Incomplete outcome data:**

1 point: The study reported complete data about the polysomnography and ultrasound abnormalities (fetal and uterine doppler).

0 points: Selective data reporting cannot be excluded.

**Table S1.** Modified Newcastle-Ottawa scoring items.

The individual components listed above are summed to generate a total modified Newcastle-Ottawa

risk of bias score for each study. Total scores range from 0 to 5.

For the total score grouping, studies were judged to be of low risk of bias (≥3 points) or high risk of bias

(<3 points).
