# Supplementary material for: Effect of the maternal sleep disturbances and obstructive sleep apnea on feto‐placental Doppler: A systematic review
Source: J Sleep Res. 2025 Jan 15;34(4):e14460. doi: 10.1111/jsr.14460 (PMC12215232; doi:10.1111/jsr.14460)
Supplement: Supplementary file 2 — TABLE S2. Risk of bias assessment. [file JSR-34-e14460-s002.docx]

| **Table 2.** Risk of bias assessment of the 4 included studies | | | | | | | |  |  |
| --- | --- | --- | --- | --- | --- | --- | --- | --- | --- |
| Author, Year | **Study design and**  **sample**  **representativeness** | **Sampling**  **technique** | **Description of**  **the**  **SBD diagnostic technique** | | **Quality of**  **population**  **description** | | **Incomplete**  **outcome**  **data** | | **Total**  **score** |
| Tang, 2010 | - | ★ | | - | | ★ | - | | ★ ★ |
| Robertson, 2020 | ★ | ★ | | - | | ★ | - | | ★ ★★ |
| Robertson, 2022 | ★ | ★ | | - | | ★ | - | | ★ ★★ |
| Onslow, 2022 | ★ | ★ | | ★ | | ★ | - | | ★ ★ ★★ |
|  | | | | | | | |  |  |
